# Co-administering Melatonin With an Estradiol-Progesterone Menopausal Hormone Therapy Represses Mammary Cancer Development in a Mouse Model of HER2-Positive Breast Cancer

**DOI:** 10.3389/fonc.2019.00525

**Published:** 2019-07-09

**Authors:** Balasunder R. Dodda, Corry D. Bondi, Mahmud Hasan, William P. Clafshenkel, Katie M. Gallagher, Mary P. Kotlarczyk, Shalini Sethi, Ethan Buszko, Jean J. Latimer, J. Mark Cline, Paula A. Witt-Enderby, Vicki L. Davis

**Affiliations:** ^1^Graduate School of Pharmaceutical Sciences, Duquesne University, Pittsburgh, PA, United States; ^2^UPMC Hillman Cancer Center, University of Pittsburgh, Pittsburgh, PA, United States; ^3^Department of Pathology, Wake Forest University School of Medicine, Winston-Salem, NC, United States

**Keywords:** breast cancer, estradiol, HER2/Neu, melatonin, menopausal hormone therapy, metastasis, progesterone receptor isoforms, uterus

## Abstract

Melatonin has numerous anti-cancer properties reported to influence cancer initiation, promotion, and metastasis. With the need for effective hormone therapies (HT) to treat menopausal symptoms without increasing breast cancer risk, co-administration of nocturnal melatonin with a natural, low-dose HT was evaluated in mice that develop primary and metastatic mammary cancer. Individually, melatonin (MEL) and estradiol-progesterone therapy (EPT) did not significantly affect mammary cancer development through age 14 months, but, when combined, the melatonin-estradiol-progesterone therapy (MEPT) significantly repressed tumor formation. This repression was due to effects on tumor incidence, but not latency. These results demonstrate that melatonin and the HT cooperate to decrease the mammary cancer risk. Melatonin and EPT also cooperate to alter the balance of the progesterone receptor (PR) isoforms by significantly increasing PRA protein expression only in MEPT mammary glands. Melatonin significantly suppressed amphiregulin transcripts in MEL and MEPT mammary glands, suggesting that amphiregulin together with the higher PRA:PRB balance and other factors may contribute to reducing cancer development in MEPT mice. Melatonin supplementation influenced mammary morphology by increasing tertiary branching in the mouse mammary glands and differentiation in human mammary epithelial cell cultures. Uterine weight in the luteal phase was elevated after long-term exposure to EPT, but not to MEPT, indicating that melatonin supplementation may reduce estrogen-induced uterine stimulation. Melatonin supplementation significantly decreased the incidence of grossly-detected lung metastases in MEL mice, suggesting that melatonin delays the formation of metastatic lesions and/or decreases aggressiveness in this model of HER2^+^ breast cancer. Mammary tumor development was similar in EPT and MEPT mice until age 8.6 months, but after 8.6 months, only MEPT continued to suppress cancer development. These data suggest that melatonin supplementation has a negligible effect in young MEPT mice, but is required in older mice to inhibit tumor formation. Since melatonin binding was significantly decreased in older mammary glands, irrespective of treatment, melatonin supplementation may overcome reduced melatonin responsiveness in the aged MEPT mice. Since melatonin levels are known to decline near menopause, nocturnal melatonin supplementation may also be needed in aging women to cooperate with HT to decrease breast cancer risk.

## Introduction

Melatonin production by the pineal gland is stimulated by darkness and inhibited by light (1). Many studies have investigated its antineoplastic properties in a variety of tumor types, including breast cancer (2, 3). Melatonin is a potent free radical scavenger but also induces responses via its MT1 and MT2 receptors. Through these receptor and non-receptor mediated responses, melatonin is reported to influence all stages of cancer, including the initiation and promotion phases of cancer development, tumor growth and progression, and metastasis (3–5). Melatonin has oncostatic effects on angiogenesis, immune modulation, tumor metabolism, oxidative stress, and apoptosis (1, 4–7). Melatonin also influences breast cancer by modulating estrogen responsiveness through its actions as a selective estrogen enzyme modulator (SEEM) to affect estrogen synthesis and as a selective estrogen receptor modulator (SERM) to inhibit estrogen receptor (ER) transcriptional activity (8).

Exposure to light-at-night (LAN) suppresses melatonin levels, induces circadian disruption, and has been correlated with multiple adverse health effects, including cancer (9). Circadian disruption in mice increases mammary cancer development (10) and, in women, LAN in night shift workers is correlated with increased breast cancer risk (11). Melatonin administration reverses the adverse effects of LAN on mammary tumorigenesis in preclinical models (12). Moreover, total blindness, which prevents suppression of melatonin by light, is also associated with a decreased incidence of breast cancer compared to blind women with light perception (13).

Nocturnal melatonin levels decline with age and reductions in the nighttime peaks result in smaller differences between the night and day levels (14). In women, the decrease in melatonin levels coincides with menopause (15–17). In addition to age, menopausal hormone therapy (HT) also diminishes melatonin secretion (18). Therefore, supplementing nocturnal melatonin levels may be necessary to reduce breast cancer risk in aging women taking HT.

In addition to its cancer protective properties, melatonin also has numerous beneficial actions on issues prevalent in aging women. The decrease in melatonin levels near menopause and its cancer-protective and other actions suggest that supplementing nocturnal melatonin levels could improve quality of life (QOL) and sleep for menopausal women and reduce aging and their risks of breast and other cancers, cardiovascular disease, atherosclerosis, osteoporosis, and diabetes (3, 17–24). However, melatonin has not been reported to relieve hot flashes (25–27).

Estrogen is the most effective therapy for reducing menopausal symptoms (28). For women without a uterus, estrogen administered alone provides symptom relief and multiple studies with estrogen-only HT report no increase in breast cancer risk with 17β-estradiol (estradiol) (29) and conjugated equine estrogens (CEE) in the Women's Health Initiative (WHI) (30, 31). However, women with a uterus need an HT that includes a progestogen to prevent endometrial cancer from unopposed estrogen exposure (32). For women with a uterus in the WHI study, the estrogen-progestin HT containing CEE and medroxyprogesterone acetate (MPA) increased breast cancer risk (33). Since these findings with CEE-MPA, HT use has decreased (34, 35), leaving many women with a uterus with the choice of no HT or less effective to ineffective options for menopausal symptom control. Most perimenopausal women have a uterus, with the CDC reporting only 10.6% of women between 40 and 44 for years 2011–2015 ever having had a hysterectomy (36, 37).

Vasomotor symptoms (VMS), which includes hot flashes and night sweats, are the most prevalent and disruptive symptoms associated with menopause. Up to 80% of women experience VMS, with varying degrees of severity and duration, which includes ~40–50 million women in the US alone (38, 39). VMS can begin 10 years prior to the final menstrual period (FMP) and decline after menopause, or begin closer to FMP with symptoms extending for many postmenopausal years (39). Moderate to severe symptoms continue in some women for over 10 years after menopause, indicating that the issues and symptoms of menopause largely remain undertreated (39–41).

For women with a uterus not taking an estrogen-based HT, the ineffective control of the symptoms that accompany the menopausal transition has profound impact on a woman's life and health. VMS reduces health-related QOL (HRQoL) and QOL, including sleep, sex, mood, cognition, and family/relationships (38, 42) and work productivity (42, 43). VMS and other menopausal symptoms are also associated with an increased risk of cardiovascular and coronary heart disease and stroke (44). Poor sleep is associated with VMS and sleep disruptions reduce QOL and HRQoL (45–47), increase cardiovascular disease risk (48), and influence mechanistic pathways in mammary carcinogenesis (49). Persistent and untreated VMS has also been associated with an increase in breast cancer incidence compared to women without VMS after long-term follow-up of women from the WHI study (50).

For women with a uterus and VMS, an estrogen-progestin HT would control symptoms but has been associated with an increased risk of breast cancer. The different WHI findings with estrogen-progestin vs. estrogen-only HT (30, 33) indicate that exposure to a progestin, like MPA, increases breast cancer risk. To diminish this risk, the natural progestogen, progesterone, has replaced synthetic progestins in many HT formulations. Administration of progesterone and MPA have differing influences on normal breast cell proliferation (51). In postmenopausal women, CEE-MPA administration significantly increased breast epithelial proliferation and mammographic breast density unlike estradiol-progesterone therapy (52). Furthermore, a meta-analysis of estradiol-based HT studies found an increased breast cancer risk with progestins, including MPA, but no increased risk with progesterone (29).

Supplementing melatonin levels may allow the use of reduced progesterone doses in HT without increasing uterine cancer risk. Melatonin has a protective effect in the uterus since night shift workers, especially obese women, have a higher risk of endometrial cancer and women with endometrial cancer have lower melatonin levels (53). Since melatonin down-regulates uterine estrogen receptors (ER) and increases uterine progesterone receptor (PR) expression (54), it has the potential to augment progesterone effects while diminishing estrogen stimulation of the uterus, despite a lower progesterone dose.

The CEE-MPA arm of the WHI study had more deaths from breast cancer and more cancers were diagnosed at an advanced stage (regional and metastatic) compared to the placebo and the CEE-only arm (55). As metastatic disease is intimately linked to breast cancer survival, it is important to first assess the safety of this HT in a preclinical model with a high incidence of metastasis. In the unactivated/protooncogene MMTV-*Neu* (*Neu*) mice, the majority of females develop primary mammary tumors that frequently metastasize to the lung (56). Tumor development occurs due to spontaneous mutations that activate the wild-type *Neu* gene (*erbB2*, HER2) to an oncogene (56, 57). Like breast cancer, carcinogenesis in this model is complex, multistep, and stochastic (56, 58). Although the resulting mammary tumors are estrogen independent, tumor development in this model requires the presence of estrogens since both tamoxifen and ovariectomy, administered prior to tumor onset, are highly effective inhibitors of oncogenesis (59–61). In women, hormones also influence HER2^+^ breast cancer since the use of HT is a risk factor for this tumor subtype (62) and, in the WHI study, the highest risk in women taking the CEE-MPA HT was attributed to HER2^+^ tumors compared to the other subtypes (55).

The cancer protective capabilities of melatonin co-administered with menopausal HT was investigated in *Neu* mice. Estradiol was selected for the HT based on its known efficacy for controlling menopausal symptoms and its improved health benefits compared to CEE, including a lower risk of cardiovascular events, reduced bone fracture risk, and improved verbal memory performance (63–67). Progesterone was included in the HT for uterine protection and due to its improved breast cancer risk profile compared to progestins, like MPA (29). In addition to using natural hormones, a low progesterone dose relative to estradiol was analyzed to minimize progestogen exposure. Nocturnal melatonin was administered to provide extra protection for the uterus from this reduced progesterone dose in addition to its potential to suppress mammary cancer. Therefore, with the goal of identifying an estrogen-based HT for effective control of VMS for peri- and postmenopausal women with a uterus that does not increase primary or metastatic breast cancer risk, the combination melatonin-estradiol-progesterone therapy was evaluated on mammary tumor development and metastatic progression in adult *Neu* female mice.

## Methods

### Animal Care

Animal procedures were approved by the Duquesne University Institutional Animal Care and Use Committee in accordance with NIH guidelines. *Neu* hemizygous study mice were bred in-house from dizygous males expressing the rat *Neu* protooncogene [FVB/N-Tg(MMTV-*Neu*)202Mul/J] (56) and wild-type FVB/N females (Jackson Laboratory, Bar Harbor, ME). Breeders and study mice were maintained on a 12:12 h light/dark cycle and fed *ad libitum* an isoflavone-free diet to prevent exposure to estrogenic soy isoflavones, which affect mammary carcinogenesis in *Neu* mice (61, 68). This control diet is a modification of the AIN-93G diet (69). The control and estradiol-progesterone diets were prepared by Harlan Teklad, Madison, WI (17β-estradiol and progesterone, Sigma-Aldrich, St. Louis, MO). Melatonin (Sigma-Aldrich, St. Louis, MO) was administered in the drinking water. The females were randomly weaned into four groups as outlined in Table 1. Treatments began at 2 months, to precede tumor initiation, and continued until the maximum age of 14 months. In a subset of mice, mammary tumor onset was assessed by weekly palpation starting at age 4 months. An additional subset of mice was euthanized in estrus at age 3 months for analysis of endpoints prior to tumor development. To adjust doses to account for the higher metabolic rate as well as the smaller size of mice, the human dose of 0.5 mg/day estradiol and 50 mg/day progesterone was provided in the diet based on dietary intake (1,800 kcal/day for women). Melatonin-treated (15 mg/L) or vehicle-treated water (0.033% ethanol) was provided during the dark cycle (18:00–06:00) and all groups were given untreated water during light hours (06:00–18:00). The estimated melatonin dose was 50 μg/night based on the measured mean nocturnal consumption of 3.5 mL water.

**Table 1:** Treatment groups for MMTV-*Neu* female mice between ages 2–14 months.

**Group**	**Diet**	**Treated drinking water (6 p.m. to 6 a.m.)^**a**^**
CON	Control diet (isoflavone-free)	Vehicle (0.03% ethanol)
MEL	Control diet (isoflavone-free)	Melatonin (15 mg/L)^**b**^
EPT	0.5 mg/1,800 kcal 17β-estradiol + 50 mg/1,800 kcal progesterone of control diet^**c**^	Vehicle (0.03% ethanol)
MEPT	0.5 mg/1,800 kcal 17β-estradiol + 50 mg/1,800 kcal progesterone of control diet^**c**^	Melatonin (15 mg/L)^**b**^

*CON, control; MEL, melatonin; EPT, estradiol-progesterone therapy; MEPT, melatonin-estradiol-progesterone therapy*.

a *Untreated drinking water was provided from 6 a.m. to 6 p.m. for all four groups*.

b *Nocturnal melatonin dose is estimated as 50 μg/night, which ~6 mg/night for women*.

c *Human doses were adjusted for mice based on caloric intake (1,800 kcal for average woman's diet). The estimated dose for mice consuming 4 g/day diet would be ~4 μg estradiol and 400 μg progesterone. The EPT diet (for the EPT and MEPT groups) contained 0.04 g/kg of blue food coloring*.

Vaginal smears, performed prior to euthanasia, were stained with Diff-Quick stain (Imeb, San Marcos, CA). Mice were euthanized by asphyxiation with carbon dioxide at age 14 months or earlier due to tumor burden, ulcerated tumors, or illness. Blood was collected and analyzed for melatonin levels as previously reported (70). Lungs were inflated with cold 4% paraformaldehyde and inspected for visible masses. Part of the mammary tissues, tumors, and uteri were flash-frozen in liquid nitrogen and stored at −80°C; the remaining sections were fixed overnight in cold 4% paraformaldehyde and stored in 70% ethanol at room temperature until processed for histopathology as previously described (69). Paraffin-embedded tissue blocks were sectioned and stained with hematoxylin and eosin (Mass Histology Service, Worcester, MA). Lung sections were examined by a board-certified veterinary pathologist (JMC) blinded to the treatment groups for determining metastatic incidence, as previously described (69).

### Mammary Gland Whole Mount Analysis

Mammary whole mounts were prepared using fixed left inguinal mammary glands, stained with carmine alum (71), and stored at room temperature in methyl salicylate (Sigma-Aldrich, St. Louis, MO). Imaging of each gland immersed in methyl salicylate was performed with a Nikon SMZ 800 dissecting microscope with an attached Olympus DP70 camera and electronic images were captured using DP control software (Olympus Optical Co. LTD. 2002). Measurements were calibrated with a metric ruler, and the parameters were quantified using Image J software in a blinded manner. Elongation of the ducts into the mammary fat pad and the length of the mammary fat pad were determined by measurement from the center of the lymph node to the farthest terminal duct and to the leading edge of the gland, respectively. Tertiary branching was determined by quantifying the number of tertiary branches per mm^2^ in three separate distal ductal areas of each gland to determine the mean number of branches per mm^2^ per mouse.

### Human Mammary Epithelial Cultures Derived From Breast Reduction Mammoplasty

Normal human mammary epithelial cell (hMEC) explants, derived from women undergoing breast reduction mammoplasty and confirmed to be disease-free histopathologically, were cultured on a thin coat of Matrigel (1:1 Matrigel: DMEM) over tissue culture plastic in MWRI medium (72). In primary culture, these cells form a complete ductal system with polarized luminal epithelial cells surrounded by myoepithelial cells, but do not form the acellular breast stroma. The JL BRL-14 explant was derived from a 22 year-old patient and maintains the ability to form pre-ductal structures in culture up to ~22 passages. JL BRL-14 cells were plated on 10 cm dishes coated with Matrigel and grown according to previously described conditions (72–75). JL BRL-14 cells were treated with 500 nM melatonin or vehicle (0.001% ethanol) in MWRI medium. After 5 h, differential interference contrast images at 200X oil immersion magnification were photographed using a Hammamatsu low light CCD camera and an FCS2 Bioptechs sealed live cell chamber with objective heater for continuous frame capture. After 28 h, the cells were harvested with trypsin, washed, and pelleted by centrifugation for assaying melatonin receptor levels by radioligand binding using 2-[^125^I]-iodomelatonin, as described below.

### Melatonin Binding

Harvested JL BRL-14 cells and tissues from the mouse mammary glands and tumors and uteri were homogenized in 1 mL ice-cold Tris-buffer with 10 μg/mL aprotinin and 1 μg/mL leupeptin (Sigma-Aldrich, St. Louis, MO) and centrifuged at 20,000 g (17,000 rpm) for 30 min at 4°C. The resuspended pellet was assayed for 2-[^125^I] iodomelatonin binding as detailed previously (76).

### Serum Collection for Hormone Assays

Whole blood was obtained via cardiac puncture at necropsy from 3-month-old female mice in estrus and transferred to ice-cold Serum Gel S/1.1 tubes (Sarstedt Inc., Newton, NC). After clot retraction, tubes were centrifugation at 16,500 g for 5 min and stored at −20°C. Serum levels of estradiol were assessed using the Double Antibody Estradiol kit (Diagnostic Products Corp., Los Angeles, CA) and levels of progesterone were assessed with the Coat-A-Count® Progesterone kit (Diagnostic Products Corp, Los Angeles, CA) according to manufacturer's protocol.

### Real-Time RT-PCR Analyses

RNA from 3-month-old mammary glands was isolated using the Absolutely RNA® Miniprep Kit (Stratagene, La Jolla, CA) according to the manufacturer's protocol and stored at −80°C. RNA purities were between 1.8 and 2.0 A_260_/A_280_ ratio. Each total RNA sample was processed with reverse transcriptase (RT) and without (no RT) using the qScript™ cDNA Synthesis Kit (Quanta BioSciences, Inc., Gaithersburg, MD) with an Eppendorf Mastercycler Epgradient (Eppendorf AG, Hamburg, Germany) for the real-time RT-PCR analysis.

The primers for the PCR reactions were designed to span at least one intron/exon boundary, whenever possible, to ensure that the amplification was not due to potential genomic DNA contamination. The primers used are as follows: *Areg* (amphiregulin) *F*: 5′-CTT TGT CTG TGC CAT CAT CC-3′ and *R*: 5′-TCC CTG AAG TAT CGT TTC CA-3′; *Erbb2* (rat erbB2 receptor tyrosine kinase 2) *F*: 5′-TGG ATG TAC CTG TAT GAG ACG-3′and *R*: 5′-GGA TTC AAG CAG CAA GGA AAG-3′; *Esr1* (estrogen receptor 1, alpha) *F*: 5′-TAT GCC TCT GGC TAC CAT TAT-3′ and *R*: 5′- CAT CAT GCC CAC TTC GTA AC-3′; *Krt18* (keratin 18) *F*: 5′-TTG CGA ATT CTG TGG ACA AT-3′ and *R*: 5′-TTC CAC AGT CAA TCC AGA GC-3′; *Ms4a1* (membrane spanning four domains, subfamily A, member one or B-lymphocyte antigen CD20) *F*: 5′-TGC CTT CTT CCA GAA ACT TG-3′ and *R*: 5′-TTG GTT GGG AAG ATA CTC CA-3′; *Pgr* (progesterone receptor) *F*: 5′-GGC AAA TCC CAC AGG AGT TTG-3′ and *R*: 5′-AGA CAT CAT TTC CGG AAA TTC-3′; PRA (progesterone receptor A from *Pgr* gene) *F*: 5′-AG TGG TGG ATT TCA TCC ATG-3′ and *R*: 5′-CTT CCA GAG GGT AGG TG-3′; *Ppia* (peptidylprolyl isomerase A or cyclophilin A) *F*: 5′-AGG TGA AAG AAG GCA TGA AC-3′ and *R*: 5′-ACA GTC GGA AAT GGT GAT CT-3′. Primers were purchased from Integrated DNA Technologies, Inc. (Coralville, IA).

The PCR reactions contained the primer pair, B-R SYBR® Green SuperMix for iQ (Quanta BioSciences, Inc., Gaithersburg, MD), and either the RT or no RT sample in duplicate. For each RNA, both an RT and no RT PCR reaction were loaded into an ABgene® plate (Thermo Fisher Scientific, Pittsburgh, PA) and run at 95°C for 3 min followed by 50 cycles of 15 s at 95°C plus 45 s at 60°C in a Bio-Rad iCycler iQ™ real-time system (Bio-Rad, Hercules CA). Melt curves were analyzed for all reactions. Data was expressed as the change in threshold cycle value (ΔC_t_) between the test and control gene, cyclophilin A (*Ppia*), from the same RT reaction. The mean ΔC_t_ ± SEM was calculated for each treatment group for statistical analyses. Fold change of gene expression as compared to the control (CON) group were determined for each treatment with the 2^−ΔΔ*Ct*^ method (77).

Prior to evaluating gene expression, RNA prepared from portions of the frozen mammary glands were prescreened with two genes to ensure the sections were similar within and between the groups. Total RNA samples were prescreened with the *Ms4a1* gene (Cd20 or membrane-spanning four-domains, subfamily A, member 1) for the absence of the lymph node since this cell-dense tissue can modify the prevalence of mammary-specific messages. Additionally, to ensure that the RNA is prepared from regions of the gland with adequate ductal structures, individual RNA samples were also pretested with *Krt18* (keratin 18). RNAs with high levels of Cd20 (lymph node-positive) or low expression of keratin 18 were excluded, and a new RNA sample prepared and pretested from another section from the mammary gland of the same animal. The treatments did not significantly modify Cd20 or keratin 18 levels by one-way ANOVA for the RNA samples used for examining expression of the tested genes (data not shown).

### Western Blot Analyses

Uteri from 14-month-old mice in diestrus were dounced on ice in 500 μL RIPA buffer with 1% NP-40 and protease inhibitors (10 μg/mL aprotinin and 1 μg/mL leupeptin). Protein concentrations were determined using BCA protein assay kit (Thermo Fisher Scientific, Pittsburgh, PA). The 7.5% polyacrylamide precast gels were electrophoresed and transferred to PVDF membranes (Bio-Rad, Hercules, CA). Membranes were blocked with 4% non-fat milk and 0.1% Tween-20 and incubated overnight at 4°C with 1:6,000 anti-ERα (ab75635; Abcam, Cambridge, MA) or 10 μg/mL anti-PR (progesterone receptor Ab-13; Thermo Fisher Scientific, Pittsburgh, PA) in PBS containing 1% ECL advance blocking agent. Membranes were the exposed to 1:20,000 goat anti-rabbit immunoglobulin (IgG)-HRP (ab6721; Abcam, Cambridge, MA), developed in the dark using ECL Plus solution (Bio-Rad, Hercules, CA). The membranes were washed and reprobed with 1:10,000 anti-GAPDH (ab9485; Abcam, Cambridge, MA) antibody and 1:5000 goat polyclonal anti-rabbit IgG (ab6721; Abcam, Cambridge, MA) for protein loading assessment. Protein bands were quantified using ImageJ software (National Institutes of Health, Bethesda, MD).

Mammary tissues and tumors were homogenized on ice with a TissueTearer^TM^ in 2 mL of 50 mM Tris, pH 7.4, and centrifugated at low speed to remove cellular debris. Samples were run in 10% polyacrylamide gels and transferred the nitrocellulose membranes. The membranes were blocked using Odyssey blocking buffer (LI-COR Biosciences, Lincoln, NE) and then treated with rabbit polyclonal progesterone receptor Ab-13 (1:1,000, Thermo Fisher Scientific, Fremont, CA) primary antibodies overnight at 4°C, treated with IRDye 800CW goat anti-rabbit (1: 20,000, LI-COR Biosciences, Lincoln, NE) secondary antibodies at room temperature for 1 h. β-actin was analyzed as a loading control with mouse monoclonal β-actin (1:10,000, LI-COR Biosciences, Lincoln, NE) and IRDye 680RD goat antimouse (1:20,000, LI-COR Biosciences, Lincoln, NE) antibodies. The membranes were analyzed using fluorescent microscopy detection at 700-channel for β-actin, and 800-channel for PR using Odyssey® Infrared Imaging System. The bands were analyzed on Image Studio^TM^ Lite software (LI-COR Biosciences, Lincoln, NE) for PRB, 116 kDa triplet form, and PRA, 81 kDa singlet form, were detected and normalized against β-Actin (45 kDa).

### Statistics

Analyses were performed using GraphPad Prism 5.0 software (GraphPad Prism Inc., San Diego, CA) with *p* < 0.05 considered significant. For analyzing the Kaplan-Meier (survival) curves, pairs of the treatment groups were compared with the log-rank test. Unpaired *t*-test (outcomes with SEM) or Fisher's exact test (incidence) was used for comparing two groups; one-way and two-way ANOVA was performed for comparisons between the four groups with one or two variables (for outcomes with SEM), respectively.

## Results

### Mammary Tumor Development and Metastatic Progression

**Figure 1:**
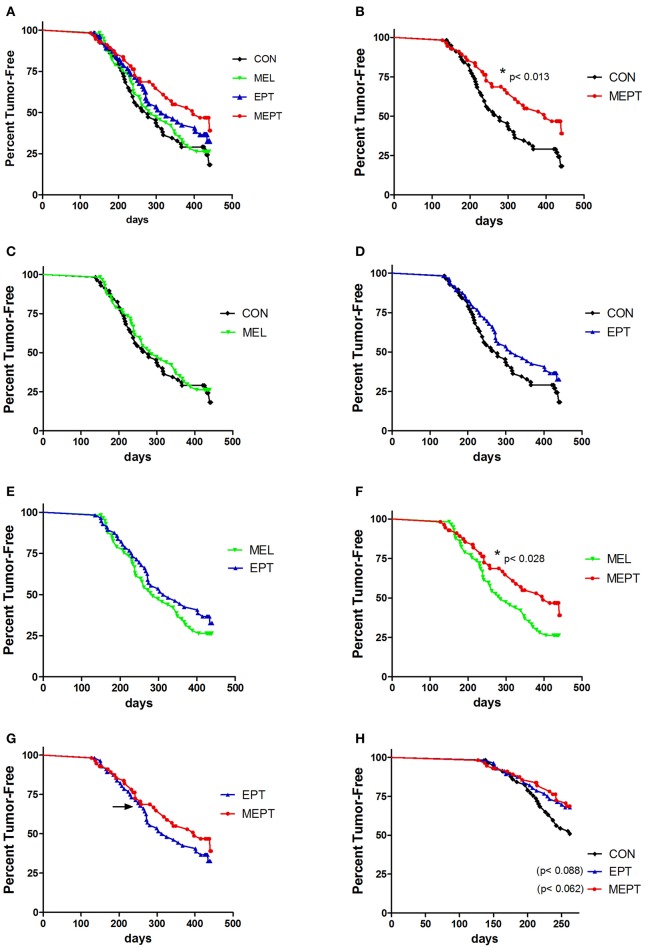
Melatonin-estradiol-progesterone therapy (MEPT) represses the development of primary mammary tumors in MMTV-*Neu* female mice. Female mice were started on the treatments at age 2 months and tumor onset was determined by weekly palpations starting at 4 months. **(A)** The Kaplan-Meier (survival) curves for all four groups are shown. **(B)** The MEPT curve (*n* = 56) is significantly different than the CON group (*n* = 57, *p < 0.013, log-rank test). **(C)** MEL (*n* = 57) and **(D)** EPT groups (*n* = 56) are not significantly different than CON mice and **(E)** are not significantly different from each other. **(F)** The MEPT curve is significantly different than the MEL group (*p < 0.028). **(G)** The MEPT curve was not significantly different than the EPT group by the Log-rank (Mantel-Cox) test. The arrow notes the age (8.6 months) when the curves separate. **(H)** The decline in tumor-free mice until age 8.6 months for EPT and MEPT mice compared to CON mice are shown (arrow in panel G denotes the maximum age used for these curves). Significance was analyzed separately for EPT and MEPT vs. CON mice, but are shown together in this graph for comparative purposes. Both treatments trended for significance for EPT vs. CON mice (*p* < 0.088, log-rank test) and MEPT vs. CON mice (*p* < 0.062, log-rank test). Group abbreviations and their treatments are outlined in Table 1.

Mammary cancer development was investigated in female *Neu* mice treated with melatonin (MEL) and the estradiol-progesterone (EPT) and melatonin-estradiol-progesterone therapies (MEPT) between 2 and 14 months of age vs. the control (CON) group (see Table 1). Kaplan-Meir curves depicting the percentage decline of tumor-free mice with age for all four groups are shown in Figure 1A. In order to analyze significance between the groups, pairs of treatments are shown in Figures 1B–G. MEPT resulted in a significantly higher percentage of tumor-free mice through age 14 months compared to CON mice (*p* < 0.013, Figure 1B). In contrast, both the MEL and EPT components of MEPT were not significantly different from the CON group (Figures 1C,D). The MEL and CON groups show a similar decline in tumor-free mice with age (Figure 1C). Although EPT separates from the CON curve with fewer mice with tumors at most ages, this difference was not significant (Figure 1D). These two components of MEPT are also not significantly different from each other (Figure 1E). However, the MEPT curve was significantly different than the MEL group (*p* < 0.028, Figure 1F), verifying that melatonin supplementation only provided cancer protection when combined with EPT.

The MEPT-induced cancer protection was not due to effects on tumor latency (Table 2), but from a reduced incidence, since significantly fewer mice developed tumors in MEPT compared to CON mice (*p* < 0.019, Table 2). In contrast, the latency and incidence of the individual MEPT components, MEL and EPT, were not significantly different than CON mice. As observed with the survival curves, tumor incidence in MEPT mice was also significantly lower than the MEL group (*p* < 0.032). These data corroborate the results from the survival curves in Figure 1 by demonstrating that only the combination therapy, but not its separate components, reduced the incidence of *Neu*-induced mammary cancer.

**Table 2:** Treatment effects on primary and metastatic mammary tumors.

**Treatment**	**Latency**	**Incidence**	**Total metastases^**a**^**	**Gross metastases^**a**^**
	**Days ± SEM**	**% mice with mammary tumors**	**% mice with lung metastases**	**% mice with gross lung metastases**
CON	252.6 ± 12.0 (*n* = 43)	75.4% (43/57)	82.0% (73/89)	52.8% (47/89)
MEL	257.0 ± 11.6 (*n* = 42)	73.7% (42/57)	76.8% (73/95)	34.7% (33/95)^*^
EPT	261.3 ± 13.4 (*n* = 36)	66.7% (36/54)	76.5% (65/85)	40.0% (34/85)
MEPT	264.2 ± 16.6 (*n* = 29)	53.7% (29/54)^*^	75.3% (58/77)	44.2% (34/77)

* *p <0.019 MEPT vs. CON and p <0.032 MEPT vs. MEL mice, Fisher's exact test for primary tumor incidence*.

*p <0.018 MEL vs. CON mice, Fisher's exact test for gross metastatic incidence*.

a *Total metastases includes gross metastases and micrometastases detected only by histopathology in tumor-bearing mice; gross metastases were visibly detected at necropsy and confirmed by histopathology*.

The MEPT and EPT survival curves overlap until age 8.6 months, but separate after this age with the MEPT curve shifting right, which indicates a cancer protective effect (Figure 1G, arrow denotes 8.6 months). When just the younger ages (until 8.6 months) were analyzed, the similar tumor development for EPT and MEPT mice trended to significance for each of these treatments compared to CON mice (Figure 1H). (The MEL group was not significantly different than the CON mice for these ages, data not shown). For ages until 8.6 months, the tumor incidence correlates with the similar curves since a lower percentage of MEPT (32%) and EPT (33%) mice developed tumors compared to the CON (49%) and MEL (46%) groups. For mice 8.7–14 months of age, the right shift of the MEPT curve is due to fewer mice developing tumors since the percentage of the remaining tumor-free mice that developed tumors in the MEPT group remained low (33%) compared to EPT (50%), CON (52%), and MEL (52%) groups These data demonstrate that only the MEPT combined therapy resulted in a continuous reduction in mammary cancer development across all the ages examined. Additionally, since tumor development in the MEPT and EPT groups was similar until 8.6 months and only MEPT was significantly different from the CON mice for all ages (*p* < 0.013, Figure 1B), the reduced tumor incidence in 8.7–14 month-old MEPT mice is essential for its curve being significantly different from CON mice. In contrast, the higher tumor incidence for EPT mice ages >8.6 months vs. ≤ 8.6 months resulted in the opposite effect, with no significant difference for all ages of EPT mice vs. CON mice (Figure 1D) and loss of the trend observed in the younger animals.

None of the treatments significantly modified the incidence of total metastases, which includes those visible (gross metastases) and not visible (micrometastases) on the lungs at necropsy, compared to CON mice (Table 2). However, the incidence of gross metastases was significantly lower in the MEL vs. CON groups (*p* < 0.018, Table 2). Fewer tumor-bearing EPT and MEPT mice had gross metastases compared to CON mice, but these differences were not significant. The reduced incidence for the visible metastases was not due to the length of tumor growth time since the days with a tumor was not significantly different between the groups (means between 103 and 119 days; data not shown).

### Mammary Gland Morphology

#### Treatment Effects on Mammary Gland Morphogenesis in Young *Neu* Mice

**Figure 2:**
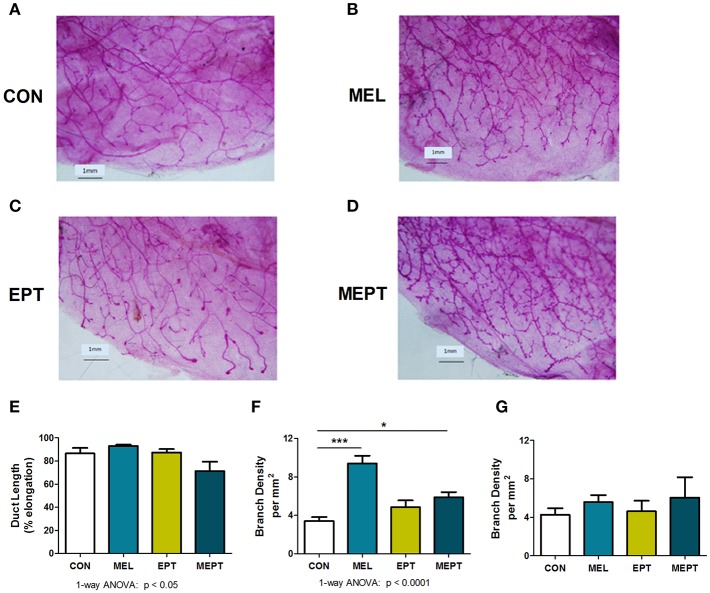
Melatonin supplementation enhances tertiary branching in young, but not old, mice. Representative photos are shown for whole mounts from **(A)** CON, **(B)** MEL, **(C)** EPT, and **(D)** MEPT mice used to analyze mammary morphology in 3-month-old mice in estrus after 1 month of treatment for panels **(E,F)**. **(E)** Ductal length (elongation of the ducts into the mammary fatpad) was significantly modified by one-way ANOVA (*p* < 0.05, *n* = 6–9), but there were no significant differences between the treatment groups and the CON mice. **(F)** The density of tertiary branching per mm^2^ were increased in the mammary glands from mice treated with melatonin for 1 month compared to CON mice (*p* < 0.0001, one-way ANOVA, *n* = 6–9). **(G)** The density of tertiary branching was similar after long-term treatment (up to 1 year) in the old mice (one-way ANOVA, *n* = 8–9). Mean ± SEM are shown. *p < 0.05 vs. CON, ***p < 0.001 vs. CON, Dunnett's multiple comparison post-test.

To assess whether mammary morphogenesis was modified by the hormone treatments, ductal elongation (regulated by estrogen), and tertiary branching (regulated by progesterone) were measured in the 3-month-old mice. Representative whole mounts are shown in Figures 2A–D. Analysis of the mean ductal length after the 1 month of treatment was significant by one-way ANOVA (Figure 2E), with no significant differences between the CON and treatment groups. However, ductal length was significantly shorter in MEPT vs. MEL (*p* < 0.05, Bonferroni post-test) when analyzed by two-way ANOVA, with a trend for the interaction of melatonin and EPT (*p* < 0.063). For tertiary branch density (Figure 2F), both groups treated with nocturnal melatonin supplementation were significantly different than CON mice (*p* < 0.001 MEL and *p* < 0.05 MEPT). When analyzed by two-way ANOVA, MEL vs. MEPT is also significant (*p* < 0.001, Bonferroni post-test) and melatonin (*p* < 0.0001) and its interaction with EPT (*p* < 0.0005) were significant. In contrast, in aged animals up to 14-months-old, there are no significant differences in tertiary branch density between the groups (Figure 2G).

#### Preductal Development in Cultured Human Mammary Explants

**Figure 3:**
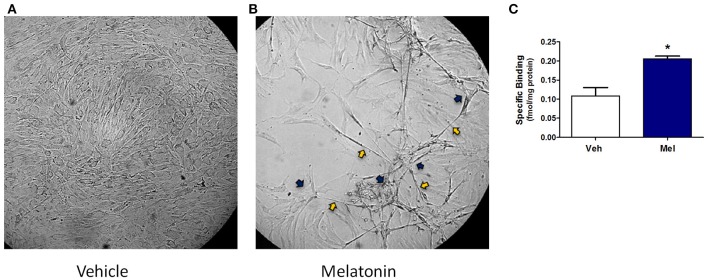
A non-diseased reduction mammoplasty explant (JL BRL-14) exposed to melatonin demonstrates accelerated pre-ductal differentiation. Preductal differentiation is manifested by the formation of double columns of elongated epithelial cells which are the precursors of tubular ducts that form spontaneously after 2–4 weeks in culture without trypsinization for this human breast explant. Photomicrographs of non-diseased JL BRL-14 explants after 5 h exposure to **(A)** vehicle (0.001% ethanol) and **(B)** 500 nM melatonin are shown (200x). The blue arrows point to cytonemes and the yellow arrows denote pre-ductal linearizations that form after 5 h in the melatonin-treated, but not vehicle-treated, BRL-14 cells. **(C)** Melatonin binding sites in JL BRL-14 explants were significantly higher after 28 h of treatment with 500 nM melatonin compared to the vehicle-exposed cells (*p < 0.05, unpaired *t*-test, *n* = 3).

Results in the adult mouse mammary glands demonstrate that melatonin supplementation influences mammary morphology. To determine if melatonin could influence similar effects in human breast cells, explants obtained from women who underwent reduction mammoplasty were investigated. In passaged explants of reduction mammoplasties, like JL BRL-14, limited differentiation occurs, including the formation of long cytoplasmic bridges between cells (cytonemes) followed by double columns of cells forming parallel to each other that develop into ducts. In cultured JL BRL-14 cells, melatonin induced the development of cytonemes and pre-ductal linearizations (double columns of cells that form and eventually become tubular ducts) within 5 h of treatment (Figure 3B); however, these structures were not evident in the vehicle-exposed cells (Figure 3A). To determine if these effects could be induced through receptor-dependent mechanisms, melatonin binding was analyzed. Melatonin binding sites were detected in both the vehicle- and melatonin-treated hMEC cultures, with significantly more receptors detected in the melatonin-exposed cells (Figure 3C).

### Melatonin Binding in Mammary Glands and Tumors

**Figure 4:**
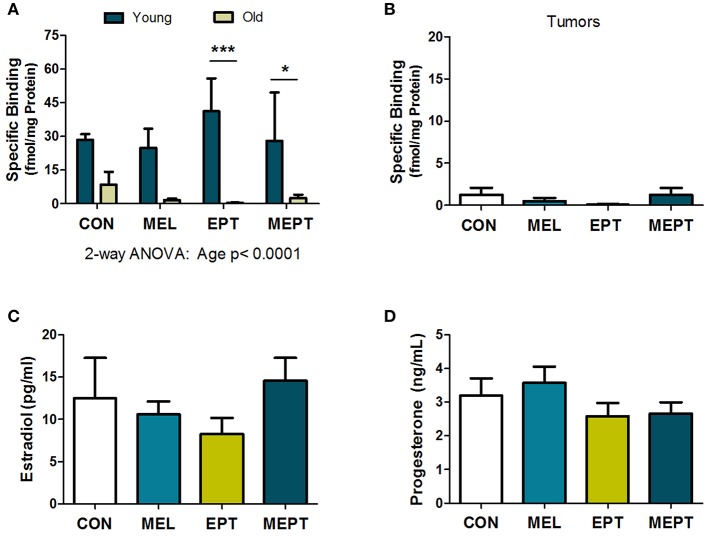
Melatonin binding sites in mammary tissues and tumors and serum estradiol and progesterone levels are not modified by the treatments. **(A)** Mammary tissues from 3-month-old mice in estrus (Young) and from aged mice (Old) in various stages of the estrous cycle (mean age ranges between 9.6 and 10.1 months) were examined for 2-[^125^I]-iodomelatonin binding. Significantly higher melatonin binding was detected in the young mammary glands (*n* = 3) compared to the old mice (*n* = 9) by two-way ANOVA (*p* < 0.0001), but treatment effects and their interaction were not significant. Significantly lower melatonin binding was detected in the old EPT vs. young EPT mice (***p < 0.001) and old MEPT vs. young MEPT mammary glands (*p < 0.05) by the Bonferroni post-test. **(B)** Like mammary tissue from aged mice, mammary tumors (age means range between 10.8 and 11.5 months, *n* = 9) had meager melatonin binding (note the lower range on the y-axis compared to **A**). No significant differences were detected by one-way ANOVA and Dunnett's multiple comparison post-test. **(C)** The treatments did not significantly modify serum estradiol levels (pg/mL) and **(D)** serum progesterone levels (ng/mL) from 3-month-old *Neu* females in estrus by one-way ANOVA. Mean ± SEM are shown.

Previous studies have reported the presence of melatonin receptors in human and mouse mammary tissues, breast tumors, and breast cancer cells (78, 79). To verify the presence of melatonin receptors in *Neu* mice, melatonin binding was examined in young mouse mammary glands prior to tumor development after 1 month of treatment (3-month-old females in estrus) and in aged animals after long-term treatment (ages 8.2–11.3 months). Melatonin binding was not significantly different by one-way ANOVA in the mammary tissues for the treatment groups compared to the CON mice for either age group. When the two ages were compared (Figure 4A), there were significantly more melatonin binding sites in the young animals compared to mammary glands from older mice (*p* < 0.0001 for age, two-way ANOVA), with binding in the old EPT and MEPT groups being significantly lower than in the young mice from the same treatment group (*p* < 0.001 for EPT and *p* < 0.05 for MEPT, Bonferroni post-test). Melatonin binding was negligible in the mammary tumors (Figure 4B), with no significant differences between the older mammary tissues and tumors by two-way ANOVA (data not shown). The treatments did not significantly modify binding irrespective of age or tissue type.

### Serum Estradiol and Progesterone Levels in Young Mice in Estrus

Treatment effects on serum estradiol and progesterone levels were examined in the 3-month-old mice in estrus after 1 month of treatment and prior to tumor onset since mammary cancer development and mammary morphogenesis can be influenced by both of these hormones. No significant differences were detected for either estradiol (Figure 4C) or progesterone (Figure 4D) levels between the treatment groups and CON mice. Since low-dose HT was administered, the lack of effect on these serum levels is not unexpected, especially with normal variations in their levels between individual mice.

### Gene Expression in Mammary Tissues Prior to Tumor Formation

**Figure 5:**
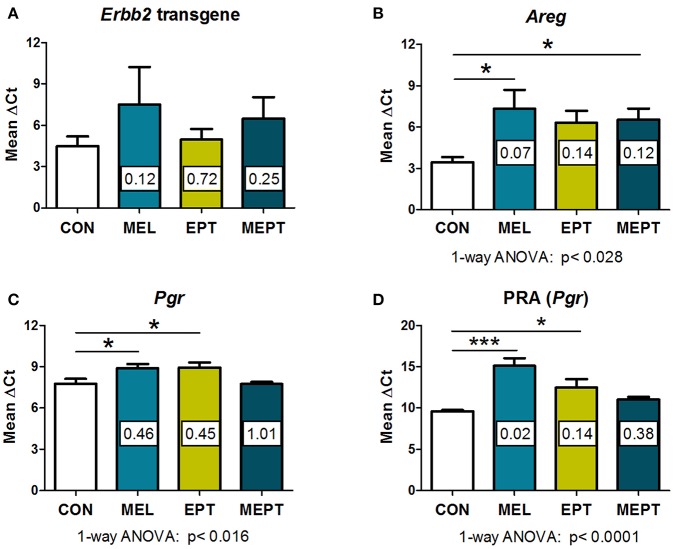
RNA expression of amphiregulin and progesterone receptors, but not the *Neu* transgene, are modified by the treatments in 3-month-old mammary tissues. RNA prepared from normal mammary tissue from female mice in estrus after 1 month of treatment was analyzed by real-time RT-PCR. The threshold cycle (C_T_) for the gene of interest was normalized to the housekeeping gene, cyclophilin A (*Ppia*), to calculate the Δ*C*_T_ values, on which the statistics were performed. The fold change of the treatment group relative to the CON group (calculated by the 2^−ΔΔ*Ct*^ method) is shown within each bar. Lower ΔC_T_ values reflect higher levels of expression. **(A)** No significant differences in the *Neu* transgene (rat *Erbb2*) levels were detected by one-way ANOVA (*n* = 3–7). **(B)** Amphiregulin (*Areg*) message levels were reduced in the groups treated with melatonin (MEL and MEPT) compared to CON mice (*p* < 0.028, one-way ANOVA, *n* = 4–6). **(C)** Progesterone receptor (*Pgr*) transcripts were lower in the MEPT group and significantly reduced in MEL and EPT mice compared to the CON group, *p* < 0.016, one-way ANOVA (*n* = 4–7). **(D)** Progesterone receptor isoform A, PRA (*Pgr* gene) was significantly down-regulated in MEL and EPT mice compared to CON mice (*p* < 0.0001, one-way ANOVA, *n* = 3–6). Mean ± SEM are shown. *p < 0.05 vs. CON, ***p < 0.001 vs. CON, Dunnett's multiple comparison post-test.

In the *Neu* transgenic model, the MMTV promoter used to express the rat *Neu* transgene has a progesterone regulatory element (80, 81). Therefore, to determine if the treatments modified transgene expression, *Neu* transcript levels were evaluated in the 3-month-old mammary tissues. No significant differences were detected between the groups (Figure 5A). These results indicate that the observed cancer protective effects are related to the MEPT treatment and not to effects on transgene expression from the MMTV promoter.

Amphiregulin (*Areg*) is an epidermal growth factor receptor (EGFR or HER1) ligand that, induces EGFR:HER2/Neu heterodimers. RNA expression of *Areg* was investigated in the *Neu* mammary glands since amphiregulin is involved in mammary carcinogenesis, is essential for early mammary morphogenesis, and is regulated by estrogen and progesterone (82–84). *Areg* RNA expression was lower in mammary tissues from the treatment groups (Figure 5B) vs. CON mice (*p* < 0.028, one-way ANOVA), but only the mice treated with melatonin (MEL and MEPT groups) were significantly different than CON mice (*p* < 0.05, Dunnett's post-test).

Expression of progesterone receptors (PR) were also examined in 3-month-old glands due to the role of progestogens in tertiary branching (85) and the elevated risk of breast cancer in the WHI study (33). PR RNA expression (*Pgr*), which includes messages for both the PRA and PRB isoforms, was significantly reduced by MEL (0.46-fold) and EPT treatments (0.45-fold) compared to CON mice (*p* < 0.05, Figure 5C). Since tertiary branching is significantly higher in MEL and MEPT mice (Figure 2F) and PRA overexpression in transgenic mice increased mammary side-branching (86), PRA expression was also investigated. PRA-specific transcripts from the *Pgr* gene were significantly decreased in the MEL (0.02-fold) and EPT (0.14-fold) mammary tissues (*p* < 0.001 MEL and *p* < 0.05 EPT), but not MEPT mice (Figure 5D). The strong downregulation of PRA messages may contribute to the decrease in total PR transcripts observed in MEL and EPT mice (Figure 5C), which did not occur in MEPT mammary tissues.

### PRA and PRB Protein Expression in the Mammary Glands and Tumors

**Figure 6:**
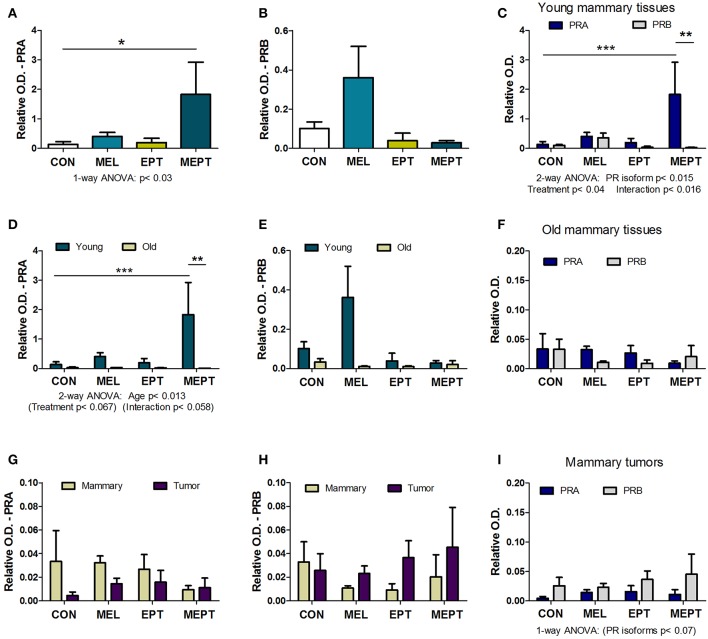
The combined melatonin-estradiol-progesterone therapy alters the balance of the progesterone receptor isoform protein expression in young mammary tissue, but not old mammary glands and tumors. PRA and PRB protein expression was determined by western blot analysis and normalized to β-actin. **(A)** PRA expression in the young mammary glands (3-month-old in estrus) was significantly elevated in MEPT treated mice compared to the CON group (*p < 0.05, Dunnett's multiple comparison post-test; *p* < 0.03, one-way ANOVA). **(B)** The PRB isoform in the treatment groups was not significantly different than CON mice by one-way ANOVA. **(C)** Comparison of expression levels of PRA and PRB in the young mammary glands shown individually in **(A,B)** by two-way ANOVA analysis demonstrated significance for the treatments (*p* < 0.04), type of PR isoform (*p* < 0.015), and their interaction (*p* < 0.016). Additionally, PRA expression in young MEPT-treated glands was significantly higher than PRA in CON mice (***p < 0.001 Bonferroni post-test; *n* = 4–9) and than PRB in MEPT mice (**p < 0.01 Bonferroni post-test). **(D)** Compared to the 3-month-old mammary tissues (Young), lower expression of PRA isoforms was detected in mammary tissues from mice with mean ages ranging between 12.9 and 14.2 months (Old) in various stages of the estrous cycle by two-way ANOVA (age *p* < 0.013), with PRA in young MEPT mice significantly different than young CON mice (***p < 0.001, Bonferroni post-test) and than old MEPT mice (**p < 0.01, Bonferroni post-test). **(E)** In contrast, comparisons of PRB expression in young and old mammary tissues was not significant by two-way ANOVA. **(F)** Unlike in the young mammary tissues, comparison for both PR isoforms in the old mammary tissues did not result in any significant differences by two-way ANOVA (*n* = 4). (Note the lower range for the y-axis for panel **F** vs. **C**). Compared to old mammary tissues, the mammary tumors excised from mice in various stages of the cycle with mean ages ranging between 8.9 and 13.7 months were not significantly different for **(G)** PRA and **(H)** PRB by two-way ANOVA. Note the reduced range for the y-axis compared to **(A,B,D,E)**. **(I)** The comparison between the two isoforms also showed no significant differences in mammary tumors, although by two-way ANOVA, the type of PR isoform approached significance *p* < 0.07, with no significance for treatment or their interaction (*n* = 3–5). Mean ± SEM are shown.

Expression of the PRA and PRB proteins encoded by the *Pgr* gene were examined in mammary tissues and tumors by western blot analysis. Prior to tumor development, unlike for its RNA, PRA protein levels in 3-month-old mice in estrus (Figure 6A) were significantly elevated (13-fold) in the MEPT-treated vs. CON mice (*p* < 0.05 Dunnett's post-test, *p* < 0.03 one-way ANOVA). However, the treatments did not significantly modify PRB expression (Figure 6B). As these two isoforms have distinct roles in the mammary gland (85, 87), the levels of PRA and PRB were compared by two-way ANOVA, which was significant for the PR isoform, *p* < 0.015; treatment, *p* < 0.04; as well as for their interaction, *p* < 0.016 (Figure 6C). Additionally, the levels of PRA protein in MEPT mammary glands was significantly higher compared to PRB levels in MEPT mice (*p* < 0.01) and to PRA levels in CON mice (*p* < 0.001, Bonferroni post-test). The dramatic difference in PRA compared to PRB levels was unique to the MEPT group with both the CON and MEL mice having similar levels of the two isoforms.

In mammary glands from mice up to age 14 months, PRA protein levels were substantially lower than in young, 3-month-old mice (Figure 6D). PRA levels for both ages were significant for age (*p* < 0.013) with trends for treatment (*p* < 0.067) and for the interaction of age and treatment (*p* < 0.058). Additionally, in young MEPT mice, PRA levels were significantly higher than in young CON (*p* < 0.001, Bonferroni post-test) and in old MEPT (*p* < 0.01, Bonferroni post-test) mammary tissues. Although PRB expression was also reduced in the older animals, no significant differences were detected by two-way ANOVA comparing the two ages (Figure 6E). Additionally, analysis of the two PR isoforms in old mammary glands by two-way ANOVA resulted in no significant differences (Figure 6F), unlike in the 3-month-old mice (Figure 6C).

No significant differences were detected by two-way ANOVA between the mammary tumors and old mammary glands for PRA (Figure 6G) and PRB (Figure 6H). The very low to no expression of PRA and PRB levels detected in the mammary tumors is consistent with previous evidence that *Neu* tumors are PR-negative (88). As with the old mammary glands, the treatments did not significantly modify expression of either isoform in the tumors, but two-way ANOVA shows a trend toward more PRB to PRA expression for the malignant tissues (*p* < 0.07 for the type of PR isoform, Figure 6I).

### Long-Term Treatment Effects on the Uterus

**Figure 7:**
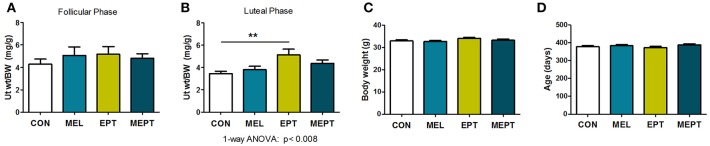
Uterine weights are elevated after long-term exposure to the estradiol-progesterone therapy but not in mice also treated with melatonin. **(A)** Uterine weights of mice were normalized to body weight after long-term exposure to the treatments for the mice in proestrus or estrus (Follicular phase) at death. Mean uterine weight/body weight (BW), mg/g, in the MEL (*n* = 50), EPT (*n* = 43), and MEPT groups (*n* = 45) were not significantly different compared to the CON group (*n* = 40). **(B)** For mice in diestrus or metestrus (Luteal phase) at death, uterine weight/body weight (BW) in EPT mice (*n* = 61) was significantly higher than the CON group (*n* = 53), unlike the MEPT (*n* = 54), and MEL (*n* = 52) groups (*p* < 0.008, one-way ANOVA). Comparison of the uterine weights for the estrogen- vs. progesterone-dominant stages (data from **A,B**) by two-way ANOVA demonstrated trends for the treatments (*p* < 0.072) and stage of cycle (*p* < 0.058), but not for their interaction, and significance for CON vs. EPT for the luteal phase, *p* < 0.05. **(C)** Body weights for the mice in any stage of the cycle (all mice in **A,B**) were similar for the four groups. **(D)** Age of death for the mice from **(A,B)** was similar for the four groups. Analysis by one-way ANOVA for all panels. Mean ± SEM are shown. **p < 0.01, Dunnett's multiple comparison post-test.

Uterine weights in aged mice were examined to determine the long-term uterotrophic effects of the treatments. No significant differences were observed for mice in estrus or proestrus stages of the estrous cycle (follicular phase) when estrogen secretion is high and progesterone levels are low (Figure 7A). However, during diestrus or metestrus (luteal phase), when estrogen levels decline and progesterone secretion increases (Figure 7B), EPT had significantly higher uterine weights (5.12 ± 0.52 mg/g; *p* < 0.01) compared to CON mice (3.44 ± 0.22 mg/g). Additionally, EPT weights in the estrogen-dominant stages (5.17 ± 0.66 mg/g, Figure 7A) were similar to the luteal phase (Figure 7B), unlike the other groups with reduced luteal-phase weights. Analysis of the data in panels 7A and 7B by two-way ANOVA trended for stage of cycle (*p* < 0.058) and treatment (*p* < 0.072), which is likely related to the decreases in uterine weight by the other groups. Body weights were similar for the groups (Figure 7C), indicating that variations in normalized uterine weights were due to treatment effects on the uterus vs. body weight. Additionally, no significant variations in age were detected between the groups (Figure 7D), with mean age of death over 1 year (treatment >10 months). These results suggest that the EPT effects with the tested estradiol:progesterone ratio were not suppressed by endogenous and/or supplemented progesterone, which resulted in continuous uterine stimulation. In contrast, melatonin addition to EPT appears to mitigate uterine stimulation since MEPT uterine weights in the luteal phase (4.38 ± 0.29 mg/g, Figure 7B) were not significantly different from CON mice and were lower than in the follicular phase (4.82 ± 0.41 mg/g, Figure 7A).

**Figure 8:**
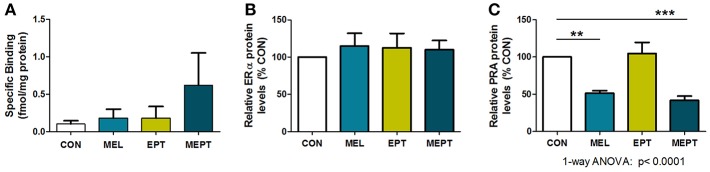
Progesterone receptor A (PRA) protein levels in the aging uteri are reduced by melatonin supplementation unlike melatonin binding sites and estrogen receptor alpha (ERα). **(A)** Uteri from 14-month-old females in each group (*n* = 5) were analyzed for 2-[^125^I]-iodomelatonin binding. Low level specific binding was detected in the uteri from all four groups with no significant differences detected by one-way ANOVA and Dunnett's multiple comparison post-test. **(B)** ERα expression by western blot analysis was determined from protein extracts prepared from uteri from 14-month-old mice in diestrus (6 μg protein/sample), and normalized to glyceraldehyde 3-phosphate dehydrogenase (GAPDH) levels. Relative ERα protein levels for the MEL, EPT, and MEPT groups based on 100% for the CON group (*n* = 4–6) were not significantly different by one-way ANOVA or Dunnett's multiple comparison post-test. **(C)** PRA expression by western blot analysis was analyzed from protein extracts (3 μg protein/sample) prepared from uteri from 14-month-old mice in diestrus and normalized to glyceraldehyde 3-phosphate dehydrogenase (GAPDH) levels. Relative PRA protein levels based on the CON group (100%,) were significantly lower in the MEL and MEPT groups compared to the CON group (*p* < 0.0001, one-way ANOVA, *n* = 5–6). Mean ± SEM are shown. **p < 0.01 vs. CON; ***p < 0.001 vs. CON, Dunnett's multiple comparison post-test.

To determine if the aging uteri remain responsive to supplemented and endogenous melatonin, estradiol, and progesterone, expression of their specific receptors was examined in 14-month-old mice in diestrus. No significant differences in uterine melatonin binding (Figure 8A) and ERα protein levels (Figure 8B) were detected. For PR, only PRA, and not PRB, was detected in the old uteri, in contrast to both receptors being measurable in 3-month-old *Neu* uteri in estrus (89). The inability to detect PRB may be related to age and/or stage of cycle (progesterone-dominant), since PR expression is estrogen-regulated. Both groups treated with melatonin (MEL and MEPT) had significantly lower PRA protein levels compared to the CON group (*p* < 0.001, Figure 8C), indicating that melatonin strongly inhibits diestrus uterine PRA levels.

## Discussion

### Treatment Effects on Mammary Tumor Development and Metastasis

Nocturnal melatonin administered with estradiol-progesterone HT significantly reduced the risk of mammary cancer. Besides requiring melatonin supplementation, the type of HT tested is likely important for the observed cancer protection, including the use of natural hormones, low dose HT, and a reduced progesterone dose. For HT alone, the similar cancer risk in old EPT vs. CON mice correlates with studies reporting no increase in breast cancer risk for natural HT (29). The lack of an HT effect on metastatic incidence in MEPT and EPT mice is likely due to the tumors in *Neu* mice being ER-/PR-negative (90) and suggests that EPT would not increase tumor aggressiveness or decrease survival in women.

CEE-MPA HT increased the risk of all breast cancer subtypes in the WHI study, with HER2^+^ cancers having the highest risk (55). In this mouse model of HER2^+^ breast cancer, the cancer protective actions of MEPT suggests that the combination therapy may also be effective for other subtypes, such as ER^+^, PR^+^, and triple negative breast cancer. But, in ER^+^ tumors, melatonin, and MEPT may also decrease tumor promotion/growth due to the antiestrogenic actions of melatonin (8). Further studies in animals and women are needed to evaluate the efficacy of MEPT in other breast cancer subtypes.

#### Melatonin and HT Cooperate to Reduce Tumor Development

Tumor development in MEPT mice was significantly reduced compared to CON mice (Figure 1B), unlike the EPT or MEL groups (Figures 1C,D), demonstrating that melatonin and HT must cooperate to suppress tumor formation. The MEPT effect was due to decreasing the incidence of mammary cancer, with no effect on latency (Table 2), indicating that the combined therapy affected tumor initiation vs. promotion. In the *Neu* mouse model, estrogens shorten the time to mammary tumor development and inhibition of estrogen action increases latency; while ovariectomy and tamoxifen treatment reduce incidence (59–61). In perimenopausal women with preexisting breast tumors, HT is reported to stimulate tumor promotion (51). However, melatonin has been reported to inhibit both the initiation and promotion phases of chemically-induced mammary carcinogenesis (90) and has estrogen inhibitory actions (8). Therefore, melatonin has the potential to modify HT effects on mammary tumor development. This potential is evident in MEPT mice since only the combination of melatonin and EPT was effective at significantly decreasing tumor incidence.

In addition to melatonin being essential for repressing cancer development, EPT is also required. The lower incidence in young EPT vs. CON mice and all ages of MEPT vs. MEL mice indicates that endogenous and supplemented melatonin, respectively, are unable to reduce mammary cancer development in mice not treated with EPT. Thus, the need for EPT to cooperate with melatonin indicates that endogenous estradiol and progesterone are not able to induce the protective melatonin-HT interaction. Perhaps the continuous delivery of estradiol and/or progesterone in intact female mice helps buffer the hormonal changes that accompany the transitions between the phases of the estrous cycle. Although EPT did not modify estrous cycling, including cycle length (89), it may modify tissue sensitivity to the hormones and/or differentially regulate responses within or outside (i.e., immune system) the mammary glands involved in carcinogenesis. The continuous EPT treatment in the mice may be similar to the use of HT in women approaching menopause since perimenopausal women continue to cycle for up to 10 years before their FMP (39) with irregular menstrual cycles and erratic hormonal changes, including hyperestrogenism (91). Thus, co-administering HT and nocturnal melatonin in perimenopausal women may also cooperate to lower breast cancer risk.

Other studies have also shown cooperation between melatonin and HT and/or estradiol. In ovariectomized rats, melatonin plus estradiol decreased uterine stimulation and improved bone formation, glycemic dysregulation, cerebral ischemia, and bladder contractility vs. the individual components (92–96). Additionally, melatonin supplementation improves several cardiovascular responses, including blood pressure, in postmenopausal women taking estradiol-MPA HT, but was ineffective in women not on HT (97, 98). Collectively, these studies show that melatonin and estradiol or HT cooperate to induce beneficial responses in women and animals, as was observed for mammary cancer risk in MEPT mice. Potentially, melatonin may also cooperate with estradiol-only HT for reducing breast cancer risk.

#### Age Effects on Tumor Development in MEPT and EPT Mice

MEPT significantly reduced tumor development compared to MEL mice for both the survival curves (Figure 1F) and incidence (Table 2). In contrast, the MEPT survival curve (Figure 1G) and incidence is not significantly different than EPT. The lack of significance is likely due to the MEPT and EPT curves and incidence being similar until 8.6 months, demonstrating that melatonin supplementation had little to no effect on tumor development in young MEPT mice. After 8.6 months of age, the right shift of the MEPT survival curve and higher percentage of tumor-free mice indicate a cancer protective effect compared to EPT mice. These results establish that melatonin supplementation is required for reducing tumor formation by the combined MEPT treatment in mice older than 8.6 months and suggest the potential benefit of melatonin supplementation in aging women taking HT.

The reasons for these age-related effects may be related to changes in melatonin and/or hormone responsiveness. For example, the need for melatonin supplementation in older mice may be related to the natural decline in endogenous melatonin secretion with age in rodents and humans (14, 99, 100). The shift to fewer mice with tumors in both treatment groups compared to CON mice, which approaches significance (Figure 1H), strongly suggests that endogenous melatonin may also cooperate with EPT in the young EPT and MEPT mice. However, after age 8.6 months, despite 12 h of darkness, this cooperation is not observed since tumor development is no longer suppressed in EPT mice; however, melatonin supplementation overcomes this deficiency in MEPT mice. Additionally, melatonin responsiveness in the mammary glands may be reduced in the aged mice due to decreased melatonin binding (Figure 4A). In fact, the ages of mice with reduced melatonin binding sites (8.2–11.3 months) correspond well with the shift in mammary cancer incidence between EPT and MEPT mice (8.6 months).

Additional reasons for the age effects of MEPT on tumor development may include changes in estradiol, progesterone, and other reproductive hormone levels, their receptor expression (i.e., the loss of the higher PRA expression in aged MEPT mice, Figure 6D), and other age-related reproductive changes, such as in estrous cycling and fecundity, which may be occurring before 8.6 months. Furthermore, other factors that influence tumor development and that are modified by age, melatonin, and/or HT may be modified prior to age 8.6 months. For example, the immune system weakens with age and both melatonin and HT improve responses associated with immunosenescence (7, 101, 102). In women, menopause is associated with the natural decline in melatonin levels (15, 16); changes in cycling, hormone levels, and fertility; as well as decreased immune function (102, 103). Further studies are needed to determine what changes are related to the age effects of MEPT and melatonin supplementation on tumor development and when they occur. Since 8.6 months indicates the age when the tumor is detected and tumor formation can occur over many weeks in mice, it would be expected that one or more of these responses change weeks prior to the 8.6 month timepoint.

Even though melatonin receptors, and potentially nocturnal melatonin serum levels, decrease in the aged mice, tumor development is suppressed by melatonin treatment in old MEPT mice. Accordingly, the supplemented melatonin levels may induce cancer protective responses within mammary-specific cells that still express melatonin receptors; in non-mammary tissues with melatonin receptors, such as immune and vascular cells (79); and/or via receptor-independent effects, such as its antioxidant activities (1).

#### Melatonin Only Effects on Tumor Development and Metastasis

Tumorigenesis was unaffected in MEL mice with equal light:dark exposures (Figure 1C), unlike in other preclinical models in which melatonin reduces mammary cancer development in animals exposed to LAN (12). These results suggest that melatonin supplementation may not provide additional protection against breast cancer for women getting long periods of complete darkness each night prior to the natural decline in melatonin production near menopause, but it may be protective for women exposed to LAN or short dark periods.

Melatonin influences many mechanisms involved in tumor metastasis, including modulation of cell-cell and cell-matrix interactions, cytoskeleton reorganization, extracellular matrix remodeling by matrix metalloproteinases, epithelial-mesenchymal transition, and angiogenesis (1, 104). The reduced incidence of gross, but not total metastases (Table 2), in MEL mice suggests that melatonin supplementation does not decrease the risk of metastasis but delays its development. The significant difference in gross metastases vs. CON mice also suggests endogenous melatonin in animals with adequate dark exposure is insufficient to delay metastatic progression. Thus, even in mammary tumors with negligible melatonin binding sites, metastatic progression may be influenced by melatonin supplementation. Its effects may occur within the tumor via melatonin receptor-independent mechanisms and/or in melatonin-responsive tumor and non-tumor tissues, such as in the immune system, blood vessels involved in intra- and extravasation, and lungs. The decreased incidence of gross metastases suggests the tumors in MEL mice may be less aggressive and at least one step in the metastatic process is inhibited, such as limiting invasion of cancer cells into the blood vessels, establishing residence within the lung, and slowing their growth and/or development to a grossly detectable size.

HT is generally stopped at diagnosis in breast cancer patients to prevent the promotional effects of estrogen, but melatonin supplementation could continue. In fact, many studies report that melatonin co-administration with chemotherapies, including tamoxifen, improves tumor regression and/or survival length with refractory cancers (1, 2, 105–109) and it improves sleep and quality of life in cancer patients and reduces chemotherapy-induced toxicity (107–110).

### Estradiol, Progesterone, and Melatonin Serum Levels

Serum estradiol and progesterone levels were not significantly modified in the EPT and MEPT groups for the 3-month-old females in estrus (Figures 4C,D). The lack of detectable differences in their levels is also consistent with the administered doses not altering estrous cycling in the young *Neu* mice, including cycle length, the number of cycles, and number of days in estrus over a 30 day period (89). However, there may be changes in their levels that were not measured, such as effects at other ages, at other times of the day/night, and at other stages or phases of the estrous cycle that may be relevant to the hormonal responsiveness in the mammary gland or other tissues/systems. For example, the increased uterine weights during the luteal phase in EPT, but not CON, mice result from modified estrogen and/or progesterone levels from the HT treatment. Therefore, the induced hormonal changes by EPT likely influence, not only the uterus, but also the mammary gland and other tissues.

Analysis of serum melatonin levels over 24 h in young, untreated *Neu* mice showed that levels fluctuate with the light:dark cycle, with significantly higher peaks 4–6 h into the dark period (70). In old *Neu* mice, daytime serum melatonin levels in MEL mice were significantly higher than CON, but not MEPT, mice. Additionally, levels in EPT mice were significantly lower than MEL mice (70), suggesting that EPT reduces endogenous melatonin levels, as previously reported in postmenopausal women taking HT (18). Nonetheless, it is unknown how age affects the melatonin levels in *Neu* mice, especially since daytime vs. nighttime levels were evaluated and day levels show less age-induced changes, unlike the nocturnal peak levels (14). In other rodent models, age is associated with reduced nocturnal melatonin levels, nocturnal to daytime levels, and melatonin receptor expression (100). In the *Neu* mice, melatonin binding sites in mammary tissues also decrease significantly with age (Figure 4A).

### Melatonin Effects on Mammary Morphogenesis in Mice and Cultured Human Cells

#### Ductal Elongation and Branching in Mouse Mammary Glands

During mammary morphogenesis, the rudimentary ducts at birth are stimulated by estrogen at puberty. This estrogen- and ERα-dependent growth continues until the ducts reach the end of the fatpad (111). In *Neu* mice, impaired ductal growth occurs due to expression of the transgene, but ERα, estradiol, and PR levels are not affected and extension of the ducts is not complete until 10–18 weeks of age, unlike the 8–10 weeks in wild-type FVB mice (112). Since treatment began near 9 weeks of age, MEL and EPT were administered before ductal growth was complete and, therefore, could potentially modify ductal length. Neither the MEL or EPT group showed a significant difference between CON mice; yet, the reduced ductal length with the combined treatment in MEPT mice again indicates cooperation between melatonin and EPT.

Melatonin supplementation significantly increased tertiary branching in the mammary glands of 3-month-old MEL and MEPT mice (Figure 2F). The variation in branching density between MEPT and MEL glands may be related to EPT decreasing melatonin levels, as previously shown in women taking HT (18) and in daytime melatonin levels in aged *Neu* mice (70).

In adults, progesterone-dependent mammary morphogenesis produces ductal side-branching (85). Unlike ductal length, this phase of mammary morphogenesis in *Neu* mice is normal since tertiary branching is unaffected (112). Studies in knockout mice for each PR isoform demonstrated that PRB is essential for mammary tertiary branching (87). In transgenic mice overexpressing either PRA or PRB, abnormal mammary morphogenesis resulted from altering the balance of these two isoforms (113), including extensive tertiary branching with PRA overexpression (88). PRA is reported to inhibit PRB, including the ability to suppress PRB-mediated mammary proliferation (114). Accordingly, altering the ratio of PRA to PRB can modify proliferation and morphogenesis in the gland. Besides direct effects of the treatments on PR expression, modifying expression of specific factors can also lead to changes in PR isoforms expression and affect mammary tertiary branching and proliferation (115, 116).

Although MEPT significantly alters the PRA to PRB balance in the mammary glands (Figure 6C), MEL mice have similar protein expression of PRA and PRB, like CON mice. Therefore, due to the highly dissimilar PRA:PRB expression in MEL and MEPT mice and similar PRA:PRB in MEL and CON mice, the balance of the PR isoforms is unlikely related to the detected increases in tertiary branching in MEPT and MEL mice. However, melatonin supplementation may modify expression of other factors that influence tertiary branching as well as progesterone responsiveness in the mammary glands via responses other than PR expression.

In aged *Neu* mice, enhanced tertiary branching in the MEL and MEPT groups is no longer detected (Figure 2G). The decreased melatonin binding in the aged mice (Figure 4A) correlates with the lack of increased tertiary branching in the older animals receiving melatonin supplementation. Thus, melatonin responsiveness is likely reduced in aged mice, which is less effective at stimulating tertiary branching. Additionally, the decline in PRA protein levels in the mammary glands with age (Figure 6D) could also influence tertiary branching. These age effects suggest that melatonin supplementation with or without HT would not influence morphogenesis in the breasts of menopausal women, the intended recipients of the combined MEPT therapy.

#### Melatonin-Induced Differentiation in Cultured Human Mammary Epithelial Cells

In the JL BRL-14 cells obtained from breast reduction mammoplasty, cytonemes, and pre-ductal structures develop within 5 h after melatonin treatment, but not in the vehicle control (Figures 3A,B). In previous studies with the untreated hMEC explants, pre-ductal structures form after several days in culture (74). Therefore, their rapid development in the melatonin-treated cells demonstrate a potent effect of melatonin on differentiation in hMEC cultures. Since the cultured hMEC explants contain multipotent stem cells, the early development of the pre-ductal structures is consistent with multiple studies that document the ability of melatonin to induce differentiation in stem cells (117–120). The effects of melatonin in JL BRL-14 cells and *Neu* mice confirm that it influences mammary morphology in both mouse and human mammary tissues. Detection of melatonin binding sites in the JL-BRL-14 cells demonstrates that the accelerated differentiation may occur, at least in part, via melatonin receptor-mediated responses.

The lower risk of breast cancer for parous vs. nulliparous women (121, 122) has been correlated with mammary gland differentiation and the resulting reduction in stem cell numbers (123, 124). However, age is important since pregnancy at early ages is protective, unlike at late ages (125, 126). Therefore, if melatonin enhances differentiation in the breasts of aging women as was observed in hMEC cultures, it would not be expected to provide cancer protection for menopausal women. Thus, the reduced mammary cancer incidence in MEPT mice suggests melatonin supplementation is acting via other mechanisms. Still, the results in cultured hMEC and *Neu* mice highlight the potential of melatonin exposure to influence mammary morphogenesis and differentiation in young girls and women, with LAN exposure and melatonin supplementation expected to have reverse effects.

### Amphiregulin Expression Related to Mammary Cancer and Morphogenesis

Previous studies report amphiregulin (*Areg*) transcription is strongly stimulated by estrogens in the mammary tissues of prepubertal ovariectomized mice (127) and by estrogen and progesterone individually and combined in adult ovariectomized rats (84). In intact mice, *Areg* expression in the mammary gland increases sharply at puberty (127) and then remains high throughout adulthood and in aged mice (128). In adult *Neu* mice in estrus, *Areg* transcripts were significantly decreased in MEPT (0.12-fold) and MEL (0.07-fold) mice compared to the CON group (Figure 5B), indicating that melatonin inhibits its expression. These data agree with previous findings that melatonin significantly repressed *Areg* mRNA levels in the mouse liver (129). Accordingly, the lower *Areg* expression in MEL and MEPT mice may result from melatonin-induced effects, such as through its antiestrogenic actions or other mechanisms.

Amphiregulin is essential for mammary gland development during puberty (83). Amphiregulin along with estrogen, ERα, progesterone, and PR are required during puberty for epithelial proliferation, ductal elongation, and terminal end bud formation in mammary gland development (130), but not earlier or later stages of mammary development, including side-branching (127). Accordingly, in the adult *Neu* mammary glands, tertiary branching and ductal elongation is unlikely to be affected by suppressing amphiregulin expression, even though melatonin supplementation affects both responses.

MEPT affected tumor initiation (incidence), but not promotion (latency) (Table 2). As an EGFR ligand, amphiregulin stimulates proliferation of mouse mammary epithelial cells and promotes mammary tumorigenesis (131). Estrogen plus progesterone-induced proliferation in normal and malignant mammary tissues is mediated through increased amphiregulin expression (84). Accordingly, reduced amphiregulin expression may decrease initiating events and, thereby, tumor incidence in MEPT by diminishing proliferation in the normal mammary epithelium. However, since amphiregulin levels are also lower in MEL mice, other factors must also be involved. Potentially, the alterations in PR isoform expression may participate since amphiregulin stimulates proliferation in PRB-expressing mammary cells (84).

Once an initiating mutation activates *Neu*, signaling occurs by heterodimerizing with EGFR in the absence of ligands like amphiregulin, unlike with the wild-type Neu receptor (132). Thus, elevated levels of amphiregulin are not necessary in mammary cells expressing an activated (oncogenic) form of *Neu* for enhanced EGFR (HER1/ErbB1) and Neu (HER2/ErbB2) signaling. Hence, amphiregulin levels are not overexpressed in the early stages of *Neu* tumorigenesis (pre-hyperplastic and hyperplastic lesions), unlike in other transgenic mouse mammary cancer models (82). Consequently, altered expression of amphiregulin would not modify Neu-induced effects on tumor promotion and progression. These actions correlate with MEPT influencing incidence and not latency (Table 2), despite lower amphiregulin expression.

In women, amphiregulin expression is elevated in human ductal carcinomas *in situ* (DCIS) compared to normal mammary tissues, as well as in primary infiltrating breast carcinomas, indicating it is an early event in human breast cancer (133). In human breast tumors, HER2/Neu may be overexpressed but is rarely mutated or activated; thus, amphiregulin expression may influence tumor promotion and progression. Higher amphiregulin expression is associated with aggressive breast cancer (134). Therefore, in women without breast cancer, if melatonin alone or combined with HT similarly reduces amphiregulin expression as in the mice, breast tissue may be less stimulated by EGFR/HER2 signaling and less prone to carcinogenesis; and, amphiregulin may influence both the initiation and promotion phases of tumor development. Thus, it would be valuable to test whether LAN and nocturnal melatonin levels and supplementation influence expression of amphiregulin in the breast tissues of aging women, including perimenopausal women taking HT.

### Altered PR Isoform Expression and Mammary Tumor Development

PRA transcript levels were significantly reduced by MEL and EPT treatments, but not by MEPT (Figure 5D). These two components also significantly suppressed *Pgr* messages; however, when co-administered in MEPT mice, *Pgr* expression was unaffected (Figure 5C). These results demonstrate that both melatonin and EPT inhibit PR and PRA RNA expression. However, these transcriptional changes are the exact opposite of the treatment effects on tumor development, in which the combination therapy resulted in significant cancer suppression.

PR transcriptional changes do not correlate with protein levels. For example, PRA transcripts were reduced in young MEPT mice (0.38-fold), but its protein levels were significantly increased (13-fold) vs. CON mice (Figure 6A). In MEL mice, PRA protein levels were higher than CON mice (2.9-fold) despite strong suppression of its message (0.02-fold). The lack of concurrence between the RNA and protein levels suggest that translational and/or post-translational regulation dictates PR expression and progesterone responsiveness in *Neu* mammary tissues, an effect that has been previously reported in the rat uterus (135).

PRA and PRB isoforms have differential actions in reproductive tissues related to the additional amino terminal section present only in PRB (85, 87). In the normal, premenopausal human breast, PRA and PRB are expressed at similar levels (136). Western blot analysis in the CON and MEL mouse mammary glands also detect similar levels for PRA and PRB (Figure 6C).

MEPT had a differential effect on PRA to PRB protein expression compared to 3-month-old mice treated with the individual components, again demonstrating that melatonin and EPT cooperate. Only MEPT mice had significantly elevated levels of PRA and little PRB, which is unlike the similar isoform expression levels in MEL and CON mice (Figure 6C). The relative expression of PRA to PRB proteins and the significantly reduced incidence of mammary cancer are both unique to the MEPT group and both demonstrate cooperation between melatonin and EPT. These PR data suggest that higher expression of PRA and its relative expression to PRB in the mammary glands may have a role in reducing cancer development in MEPT-treated mice.

Studies suggest that PRB mediates proliferation in mammary epithelial cells since PRB cells co-localize with a proliferation marker (BrdU) in pregnant animals (137) and in ovariectomized adults treated with estradiol and progesterone (84). Additionally, proliferation and differentiation are normal in the mammary epithelium of mice expressing only PRB, unlike in mice expressing only PRA (87). Inhibition of PRB-mediated proliferation by PRA also suggests that a higher relative expression of PRA:PRB may result in suppressed proliferation and mammary cancer development. Therefore, associations between equivalent expression of both isoforms in MEL and CON mice without mammary cancer protection and elevated PRA:PRB in MEPT mice with reduced cancer risk suggest that the combination therapy modifies the balance of the PR isoforms to a more favorable profile for diminishing mammary tumor development.

Unlike 3-month-old mice, aged MEPT and CON mice have similar PRA protein levels (Figure 6D). Age likely affects its expression since all groups show a reduction in PRA levels in the old vs. young mammary glands (Figure 6D). Other studies report that, with age, PR expression changes from uniform to scattered in the mammary glands of adult animals and proliferation becomes infrequent (89). Additionally, PR expression in the mammary epithelium of older mice is significantly lower compared to 14-week-old females (138) and PRA levels are significantly lower by age 5 months compared to 3-month-old mammary tissues (137). The decline in PRA levels in *Neu* mammary glands with age (Figure 6D) may be affected by melatonin responsiveness since melatonin binding is significantly lower in older mammary glands (Figure 4A). Therefore, melatonin supplementation may be unable to coordinate with EPT to increase PRA in aging MEPT mice.

Amphiregulin expression is significantly decreased in the mammary glands of young MEPT mice. Proliferation is induced by amphiregulin and progesterone primarily in epithelial cells expressing only PRB, and not those expressing ERα, PRA, and PRB (84). Potentially, suppressed amphiregulin expression in MEPT mice along with the elevated relative expression of PRA:PRB may contribute to the lower percentage of developing tumors. However, amphiregulin and PR levels alone do not fully explain the tumor outcomes, such as in MEL mice. As cancer development is complex, additional factors that are differentially regulated by the combination of melatonin and EPT likely also contribute to reducing tumor development.

### Hormone Treatments Effects on the Uterus

Melatonin binding analyses indicate that the uteri remain melatonin-sensitive through age 14 months (Figure 8A). Previous studies report that melatonin inhibits estrogen stimulation of the uterus since LAN accelerates spontaneous uterine carcinogenesis in mice (139) and co-administration of melatonin and estradiol in ovariectomized rats decreases endometrial thickness and prevents atypia compared to the estrogen-only group (93). In women, a higher risk of endometrial cancer is also associated with lower levels of melatonin and night shift work (53). MEPT mice, with melatonin supplementation, did not have the significant increase in uterine weight detected in EPT mice. Thus, endogenous melatonin appears insufficient, even with ample dark exposure, to prevent uterine overstimulation after prolonged exposure to EPT in the aged mice (Figure 7B). Since melatonin supplementation corrects this issue and melatonin binding sites are detected in the uterus, these data suggest that endogenous melatonin levels in the aged animals are inadequate for reducing EPT-induced uterine stimulation. Both the uterotrophic and mammary tumor data in EPT vs. MEPT mice indicate that supplementing endogenous melatonin levels is protective in aged mice.

Melatonin may protect the uterus by suppressing estrogen action, such as by downregulating ER and/or increasing PR expression (54). Although the treatments did not reduce ERα expression in the 14-month-old uteri in diestrus (Figure 8B), modifications at other ages or stages of the cycle may help protect MEPT uteri. Reduced PRA expression in MEL and MEPT uteri (Figure 8C) demonstrates a melatonin-protective effect, since PRA overexpression in transgenic mice is associated with endometrial hyperproliferation, hyperplasia, and atypia (140). Additionally, the lack of effect of EPT on uterine PRA expression correlates with its chronic uterine stimulation. Since both MEL and MEPT suppressed PRA levels, nocturnal melatonin supplementation may also repress uterine PRA in untreated and HT-treated perimenopausal women.

Although their use in HT elicits concerns about breast cancer risk, progestins prevent uterine hyperplasia and endometrial cancer by combating estrogen stimulation. During perimenopause, progesterone secretion declines with continuing and often excessive estrogen production (91). In postmenopausal women, obesity also elevates systemic and local estrogen levels and the risk of endometrial cancer (141). In EPT-treated mice, progesterone did not increase mammary cancer risk (Figure 1D), but its dose was insufficient to inhibit uterine stimulation during the luteal phase of the estrous cycle (Figure 7B). However, in MEPT mice, melatonin supplementation significantly decreased mammary cancer risk without overstimulating the uterus to further support the previous reports of melatonin's uterine protective effects (53, 93, 139). Therefore, combining melatonin with a progesterone-containing HT resulted in reduced uterine stimulation in addition to mammary cancer protection. However, the choice of using progesterone instead of a progestin may be critical since progestins increased breast cancer risk (29) and MPA has more proliferative effects than progesterone in the breast (51) and mouse mammary glands (142).

Recently, the HT tested in this study was investigated in a randomized, placebo-controlled clinical trial, but without melatonin supplementation. The oral estradiol (0.5 mg/day) and progesterone (50 mg/day) doses tested herein (doses adjusted for mice) were investigated in the REPLENISH study for endometrial hyperplasia and control of VMS. This HT dose did not result in endometrial hyperplasia after 12 months in postmenopausal women (143), unlike the increased uterine stimulation in the luteal phase of EPT mice. Plus, this estradiol-progesterone HT significantly reduced the frequency and severity of VMS (143) and resulted in significant improvements in QOL and sleep in women with moderate to severe VMS (144, 145). The results from this clinical trial indicate that the HT tested in the *Neu* mice would be effective for treating menopausal symptoms, increasing QOL, and protecting the uterus in peri- and postmenopausal women. Future studies are needed to evaluate the benefits of co-administering nocturnal melatonin with this and other HTs, but results in the MEPT mice suggests the combination melatonin-estradiol-progesterone HT also has the potential to reduce breast cancer risk.

### Summary

Co-administering nocturnal melatonin with a natural, low dose HT significantly repressed the development of mammary cancer in MEPT mice, unlike its individual components. Although the similar risk of mammary cancer in EPT and CON mice correlates well with studies in menopausal women reporting no increased risk of breast cancer with estradiol-progesterone HT (29); the addition of melatonin to this HT results in cancer protection in the MEPT mice. MEPT significantly reduced tumor incidence, but not latency, indicating that the combination therapy inhibits tumor initiation. The separation of the MEPT and EPT survival curves and lower tumor incidences in MEPT vs. EPT mice after age 8.6 months verify that melatonin supplementation is essential for cancer protection in aging MEPT mice. Collectively, these data indicate that melatonin and HT cooperate to suppress mammary cancer development and suggest that melatonin supplementation in aging women taking HT may also reduce their breast cancer risk. After tumor formation, melatonin supplementation reduced the risk of gross metastases in MEL mice. In mammary glands prior to tumor development, melatonin supplementation alone and combined with HT reduced amphiregulin expression and increased tertiary branching in MEL and MEPT mice. As with tumor development, melatonin, and EPT cooperate to increase PRA expression and PRA:PRB balance in the normal mammary tissues and reduce ductal elongation only in MEPT mice. The MEPT-induced changes in amphiregulin and PR isoform expression may contribute to its cancer protective capabilities. Future studies are needed to evaluate the potential benefits of nocturnal melatonin supplementation on breast cancer risk in women taking HT and to compare the efficacy of MEPT on other breast cancer subtypes, such as ER^+^, PR^+^, and triple negative breast cancers.

## Data Availability

This manuscript contains previously unpublished data. The name of the repository and accession number are not available.

## Ethics Statement

Animal procedures were approved by the Duquesne University Institutional Animal Care and Use Committee in accordance with NIH guidelines.

## Author Contributions

PW-E and VD designed and supervised the study. BD, CB, MH, WC, KG, MK, SS, EB, JL, JC, VD, and PW-E performed the research. BD, CB, MH, KG, JL, PW-E, and VD analyzed the data. BD, CB, PW-E, and VD wrote the article. All authors reviewed the manuscript.

### Conflict of Interest Statement

PW-E and VD are inventors of US Patent 8618083 (2013) and 9370526 (2016). The remaining authors declare that the research was conducted in the absence of any commercial or financial relationships that could be construed as a potential conflict of interest.

## Abbreviations

CEE: conjugated equine estrogens
CON: control
EGFR: epidermal growth factor receptor
estradiol: 17β-estradiol
EPT: estradiol-progesterone therapy
ER: estrogen receptor
FMP: final menstrual period
HER: human epidermal growth factor receptor
hMEC: human mammary epithelial cell
HRQoL: health-related quality of life
HT: hormone therapy
LAN: light at night
MEPT: melatonin-estradiol-progesterone therapy
MEL: melatonin
MPA: medroxyprogesterone acetate
QOL: quality of life
PR: progesterone receptor
VMS: vasomotor symptoms.
